# Anti-corruption and bank performance: Evidence from a socialist-oriented economy

**DOI:** 10.1371/journal.pone.0292556

**Published:** 2023-10-09

**Authors:** Thang Xuan Nguyen

**Affiliations:** Graduate School, National Economics University, Hanoi, Vietnam; Lincoln University, NEW ZEALAND

## Abstract

This current study aims to investigate the impact of anti-corruption on bank performance in Vietnam, an emerging socialist country with a high level of perceived corruption. An increasing number of financial frauds and corruption have been made public in Vietnam as a result of a vigorous anti-corruption drive in the country. Using a news-based approach to measure anti-corruption in Vietnam, the results of our empirical analysis suggest that anti-corruption has a positive impact on the profitability of Vietnamese commercial banks, however, it manifests in the long term. We do not find an immediate impact of anti-corruption on overall bank performance, but we find that bank profit per branch increases under intensified anti-corruption in the country, suggesting that optimizing branch structure is important to Vietnamese bank under uncertainty.

## 1. Introduction

In recent years, Vietnam has undergone an intensive crack down on corruption that results in large-scale investigations and prosecutions of thousands of government officials, a large number of top-tier and high-rank politicians and high-profile agents in public firms. Previous studies show that anti-corruption exerts substantial economic impacts on firm-level operations and decision-making, however, mostly concentrate on studying the anti-corruption activities in China [1–3]. As the high-profile anti-corruption campaign of Vietnam attracts attention from not only Vietnamese but also observers from outside of the country, the economic impact of such an unprecedented event is, surprisingly, under-researched.

The literature of anti-corruption in Vietnam is embryonic and seems to be correlated with the increasingly intensive anti-corruption campaign in the country. Previous studies in this line of literature show that anti-corruption has significant economic impacts on firm growth [4], non-financial firms’ performance [1, 5], and corporate investment [6]. The literature solely focus on how the anti-corruption campaign exerts its economic impacts on non-financial firms’ outcomes and do not cover the impact of anti-corruption on the banking sector of Vietnam, a sector that exposed to various corruption scandals in the countries during the most recent decades. The banking sector traditionally hold a dominant position in the Vietnamese financial system. Most financial institutions in Vietnam are banks, and they have extensive branch networks across the country. This setting makes bank loans a convenient choice for firms seeking capital. The government has active supporting policies and encourages the role of commercial banks in the economy, often considering them key partners in development initiatives. Such a policy approach has further strengthened the central role of the banking sector in the economy.

There is a series of banking regulation violation and depletion of bank assets in a number of Vietnamese commercial banks, for examples, Agribank during 2012–2016, Asia Commercial Bank during 2012–2014, Global Petro Bank during 2014–2015, Ocean Bank and Vietnam Construction Bank 2014–2018, Trust Bank during 2017–2023, among others. The country’s transition from a centrally planned economy to a socialist-oriented market economy has led to distinct challenges and opportunities in addressing corruption, for example, a decentralized power system [7], a dominance public sector in the economy, and a strong banking system that contributes about 16 to 18 percent to the country’s annual GDP [8]. Given the gap in the literature and the importance of the banking sector in the economy of Vietnam, it is compelling to study how the anti-corruption campaign of Vietnam influences financial performance of Vietnamese banks. Given that anti-corruption might improve the business environment and the institutions of the country, we predict that the anti-corruption campaign enhances bank performance in Vietnam.

Using a sample of Vietnamese commercial banks during 2005–2019, we empirically test the economic impact of anti-corruption on bank performance. To measure anti-corruption intensity, we follow Hoang et al. [5] to use the frequency of news on major Vietnamese online newspapers that mention anti-corruption policy and activities of the government. To evaluate bank performance from different perspective, we use several measures of bank performance, including average branch profit, profit per employee, and the conventional one–returns on assets of banks. We use the fixed effect estimator with control for confounding effects at firm- and macro-level. The empirical analysis yields some interesting findings. We do not find an immediate impact of anti-corruption on overall bank performance; however, we document that average bank branch profitability increases with anti-corruption, suggesting that Vietnamese commercial banks optimize their profitability at branch-level under the changes in the institutions of the country. When looking at bank performance for a longer period, we find that bank performance, regardless of performance measurements, improves significantly after two years following the increased anti-corruption intensity. This is further evidence of the anti-corruption campaign in Vietnam enhancing the business environment and institutions of the country, hence, exerting a lagged positive impact on bank performance. Our findings support the “grabbing hand” feature of corruption in Vietnam that corruption hinders economic growth, therefore, anti-corruption efforts can mitigate the adverse impact of corruption and enhance the financial performance of the banking sector in Vietnam.

This current study contributes to the literature in three specific ways. First, our findings shed light on the economic impact of anti-corruption on the banking sector. Our findings are novel and unique, especially our measurement of anti-corruption is rooted in the emerging textual-based approach in contemporary economic and finance literature. Given that previous studies generally employ event study settings [9–11], we contribute to the literature by applying a newly developed measure of anti-corruption to arrive at the dynamic impact of anti-corruption on bank performance in Vietnam. As anti-corruption always occurs during the history, event study designs may not fully capture the up-and-down in the developments of anti-corruption activities of the government. Our research design overcomes this limitation and suggests the use of a measurable variable for empirical analysis of the impact of anti-corruption on economic outcomes. Second, we complement corruption literature by adding empirical evidence from the anti-corruption perspective. Given that most studies in the field focus on the impact of corruption, be it in Vietnam [12–15], China [16–18], India [19], or in developed countries [20], our findings add to the understanding of how public efforts on “reversing corruption” economically work. Third, our study provides one of the first references for the impacts of anti-corruption on bank in the context of Vietnam, an emerging socialist country with a high level of perceived corruption according to the Corruption Perception Index provided by Transparency International. The banking sector has a crucial role in the financial system of Vietnam and link tightly to the changes in government policy, cracking down on corruption, and macroeconomic conditions. Therefore, our findings on the impact of anti-corruption on bank performance provides a reference to policymaking and future research in this field to evaluate the effectiveness of the anti-corruption policy of Vietnam.

The rest of the paper is as follows. Section 2 reviews related literature and develop research hypothesis. Section 3 introduces the research design and data. Section 4 reports and discusses the empirical results. Section 5 provides implications and concludes the study.

## 2. Hypothesis development

Corruption is a pervasive issue that undermines economic development [21–23], erodes public trust [24], lowers equality and fairness [25, 26], and causes financial instability [27, 28]. Within the context of the banking sector, corruption poses significant risks and challenges, affecting the performance and stability of banks [29–32]. In this vein, anti-corruption attempts of the government, depending on the intensity and stringency of the actions, may alleviate those negative impacts of corruption. However, currently literature only focusses on the impact of bank-level anti-corruption disclosure on bank performance [33] or the impact of government’s anti-corruption on non-financial firms’ performance [1, 5]. The understanding of how government’s anti-corruption influences bank performance remains ambiguous.

Previous studies in the field show that anti-corruption has significant influence on non-financial firms’ performance [1, 5], corporate research and development [3], corporate investment [6, 11], and financial reporting quality [9]. While the majority of the studies in the field concentrates on studying the impact of anti-corruption on non-financial firms, only an insignificant volume of literature investigates the topic from the lenses of the banking systems. A recent study by Nobanee et al. [33] suggest that less anti-corruption disclosure at bank-level in Abu Dhabi stock exchange and Dubai financial market matters to bank performance, but the authors do not examine anti-corruption at the macro-level. The authors demonstrate that anti-corruption disclosure to central banks and regulators has an immediate negative impact on the performance of different bank groups, including conventional banks and Islamic banks. However, the authors do not go further to see whether such a negative impact can be sustained in the longer period. As bank-level anti-corruption may be strongly correlated to the government’s anti-corruption efforts, there may exist a linkage between firm performance and macro-level anti-corruption as a government policy.

Theoretically, intensive and stringent anti-corruption policy by the government may reduce corruption, and thus improve the institutional quality and the economic system of the country in several way. First, government anti-corruption measures contribute to creating a more transparent and predictable business environment. When corruption is effectively addressed, it reduces the costs and uncertainties associated with unethical practices, benefiting banks and facilitating long-term business growth and performance [34]. Second, stringent anticorruption policy help strengthen the rule of law, ensuring fair and impartial judicial systems and law enforcement. When the rule of law and governance are upheld, it provides a strong legal framework for banks to operate, protecting their rights, contracts, and investments [35]. This enhances a transparent business environment that positively impacts long-term bank performance. Third, a strong commitment to anti-corruption by the government encourages investor confidence and trust in the financial system. Foreign and domestic investors are more likely to trust and invest in a jurisdiction with robust anticorruption measures [36]. As such, anticorruption may stimulate investment and ensure stability, through which it exerts a long-term positive impact on bank performance.

Following the above discussion, we expect that anti-corruption may enhance bank performance in the long-term. Our research hypothesis is as follows:

**Hypothesis 1**: Anti-corruption exerts a positive impact on bank performance.

There is a note that the literature of corruption in Vietnam mainly focus on the impact of corruption [4, 12–15], but not the impact of anti-corruption. One of the reasons for this shortcoming may be attributable to the limited data on corruption and anti-corruption activities of Vietnam available for research. Since Hoang et al. [5] developed a news-based index of anti-corruption using textual data from Vietnamese major online newspapers [5, 6], empirical investigation of the impact of anti-corruption on corporate outcomes and economic activities become more feasible.

## 3. Research methodology and data

### 3.1 Empirical model and variables

We use the following model to investigate the impact of anti-corruption on bank performance:

$${Profitability}_{i,t}=\alpha +\beta {Anticorruption}_{t-1}+\sum Control+\;\gamma_{i}+\varepsilon_{i,t}$$ (1)

where *Profitability*_*i*,*t*_ is the measurement of bank *i*’s performance in year *t*; *Anticorruption*_*t*−1_ is the measure of anti-corruption of the country in year *t-1*; ∑ *Control* is the vector of control variables at firm- and macro-level; *γ*_*i*_ denotes the bank-fixed effect; *ε*_*i*,*t*_ is the error term of the model. We do not include time-fixed effect because our anti-corruption variable (which is time-variant and does not vary in the cross-sections of banks) would be strongly correlate with time fixed effects. Therefore, the inclusion of time fixed effects would drain the explanatory power of the anti-corruption variable, which is our variable-of-interest. Instead of doing so, we cluster standard errors by year, so that the model is more appropriately specified [6].

We use three different approaches to measure bank profitability. First, we use the conventional measure of firm profitability with return-on-assets (ROA) and return-on-equity (ROE) as in previous studies [18, 37]. We do not use Tobin’s Q as the measure of bank performance because a large number of banks in Vietnam are not listed on Vietnam stock markets, thus data on market value of those banks are not available. Second, we use the ratio of profit after tax scaled by the number of bank branches (NW_PROFIT) as another measure of bank performance relative to its branch structure. Third, we scale profit after tax by number of employees of the bank during the year (EMP_PROD) as a measure of bank profitability taken average employee productivity into account. Again, banks with higher profit per employee means they have higher overall employee productivity. Using those three approaches to measure bank profitability, we attempt to address the relation between anti-corruption and bank profitability from different perspectives.

To measure anti-corruption intensity of Vietnam, we use the news-based anti-corruption index of Vietnam proposed by Hoang et al. [5]. The authors employed Python text mining programing to count the number of Vietnamese articles about anti-corruption in Vietnam from three major online newspapers of Vietnam (Vnexpress, Dantri and Nhandan) during 2005–2019. The content of those articles must contain information about anti-corruption activities such as investigation, prosecution, or anti-corruption regulation amendments in the country. This approach is similar to that of Qu et al. [38] when counting the number of news articles on corruption in Chinese major newspapers, however, Hoang et al. [5] focus only on anti-corruption efforts of Vietnam. By scrapping and filtering anti-corruption keywords from 129,154 news articles in the three Vietnamese major online newspapers during 2005–2019 [6], the authors provide a monthly anti-corruption index for public use. As the index measures anti-corruption by the number of news articles about anti-corruption activities, we can capture not only the real legal cases of corruption convictions but can also capture public attention on anti-corruption activities performed by the government. We annualize the index by taking the mean of the monthly index during a financial year, then log-transform the annualized index to rescale the variable. We name the variable ACINDEX.

Following previous studies in the literature of bank performance [19, 37, 39], control variables used in Model (1) include bank size (the logarithm of bank’s total assets–BANKSIZE, and the logarithm of the number of employees of the bank–EMPSIZE), equity-to-assets ratio (ETA), Non-performing loans ratio (NPL), loan loss provision ratio (LLP), and macro-economic factors such as Gross Domestic Product growth (GDP), inflation (CPI) and real interest rate (RIR). All bank-level control variables are lagged by one period in our regressions.

### 3.2. Data and sample

**Fig 1:**
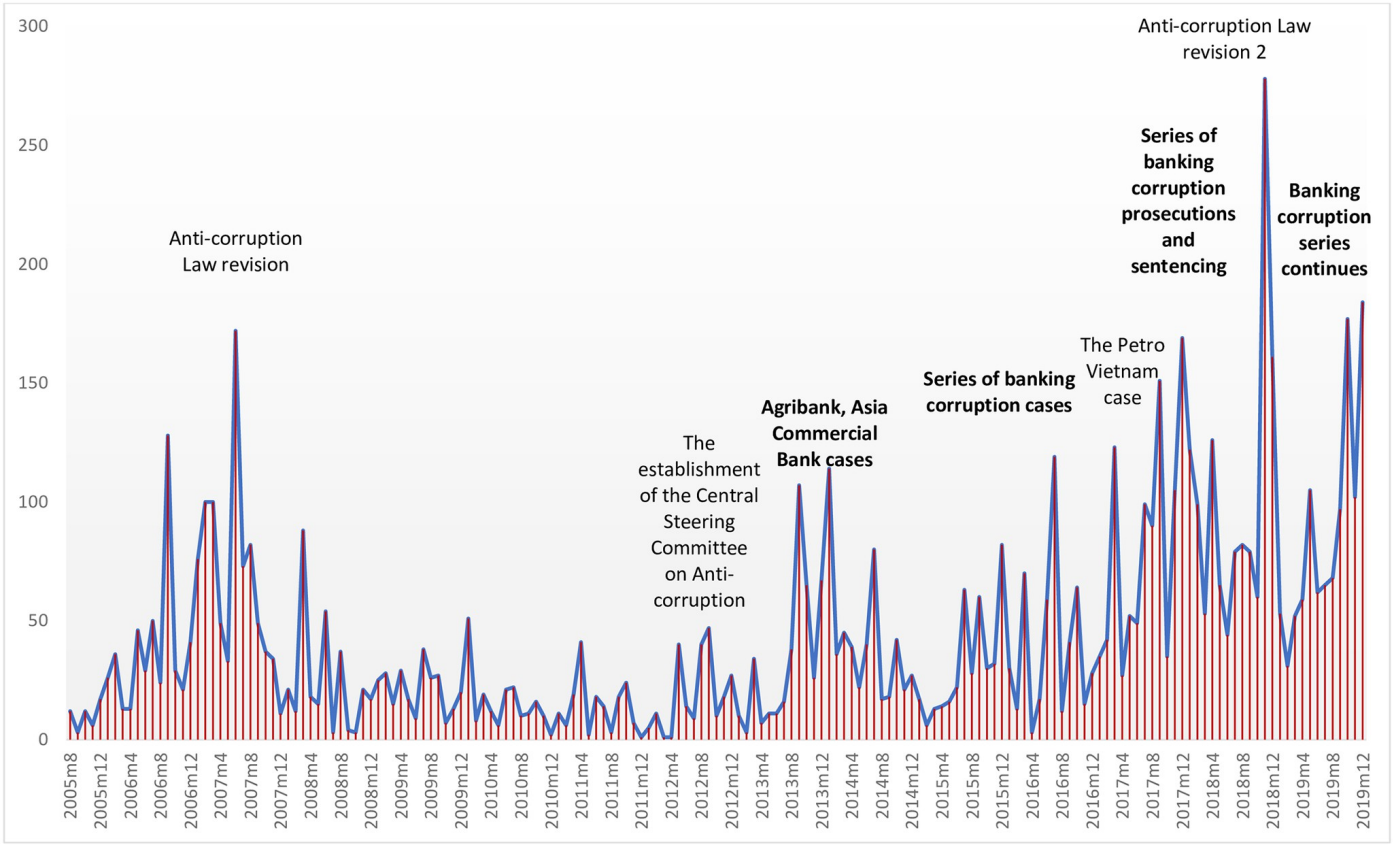
The developments of the anticorruption campaign in Vietnam.

Our sample covers Vietnamese commercial banks with available data during the period from 2005–2019. Bank data is provided by Le et al. [40]. Macroeconomic data is from World Bank’s open database. The news-based anti-corruption index data and its composite indexes are from Hoang et al. [5] ‘s GitHub file repository. After excluding all missing values in our data, we obtain a final sample consisting of 337 bank-year observations of 41 Vietnam commercial banks during 2005–2019. There is an important note that six out of those 41 banks had merged with other commercial banks during the study period, leaving the number of operating banks at 35 at the end of 2019. All continuous variables are winsorized by the 1^st^ and the 99^th^ percentiles to alleviate the potential impact of outliers on our empirical outcomes. Table 1 presents all variable descriptions and summary statistics; Table 2 shows the pairwise correlation matrix of variables used in Model (1). Fig 1 presents the plot of the anti-corruption index [5] with description of related events.

**Table 1:** Variable summary.

Variable	Description	N	Mean	Standard deviation	Min	Max
NW_PROFIT	Profit after tax scaled by number of branches of the bank, then scaled by 1,000	431	10.367	18.145	0.001	132.928
EMP_PROD	Profit after tax scaled by number of employees of the bank, then scaled by 1,000	466	0.256	0.251	0.001	1.471
ROE	Profit after tax scaled by total shareholders’ equity	627	12.526	9.399	-5.100	45.100
ROA	Profit after tax scaled by total assets	623	1.275	1.078	-0.420	5.380
VNEINDEX	The log-transformed anti-corruption index that counts the number of online news articles about anti-corruption in Vietnam using data from Vnexpress.net.	507	4.859	0.550	4.248	5.858
DTINDEX	The log-transformed anti-corruption index that counts the number of online news articles about anti-corruption in Vietnam using data from Dantri.vn	507	4.543	0.888	3.091	6.157
NDINDEX	The log-transformed anti-corruption index that counts the number of online news articles about anti-corruption in Vietnam using data from Nhandan.vn	507	3.935	0.524	3.296	4.984
ACINDEX	The log-transform anti-corruption index that counts the total number of online news articles about anti-corruption in Vietnam using data from all three online newspapers combined.	507	5.660	0.611	4.804	6.681
BANKSIZE	Log of total assets of the bank	627	17.726	1.715	12.334	21.088
ETA	Equity-to-total assets ratio of the bank	627	11.433	7.921	2.450	46.240
NPL	Non-performing loans scaled by total loans of the bank	577	1.882	1.703	-1.120	11.400
LLP	Loan loss provisions scaled by total loans of the bank	603	1.237	0.847	-0.490	5.260
EMPSIZE	Log of the number of employees of the bank	468	8.048	1.326	4.554	10.552
GPD	Gross Domestic Product growth rate (in %)	14	6.515	0.681	5.400	7.500
CPI	Consumer Price Index annual change rate (in %)	14	7.876	5.878	0.600	23.100
RIR	Real interest rate (in %)	14	0.552	7.212	-20.500	9.000
ELECTION	Dummy variable that equals one if the year is the year of legislative election in Vietnam, zero otherwise	14	0.165	0.371	0.000	1.000

**Table 2:** Correlation matrix.

Variables	(1)	(2)	(3)	(4)	(5)	(6)	(7)	(8)	(9)	(10)	(11)
(1) NW_RPOD_t_	1.000										
(2) EMP_PROD_t_	0.593***	1.000									
(3) ACINDEX_t-1_	0.150***	0.053	1.000								
(4) BANKSIZE_t-1_	0.375***	0.265***	0.216***	1.000							
(5) ETA_t-1_	-0.063	0.040	-0.148***	-0.563***	1.000						
(6) NPL_t-1_	-0.137***	-0.231***	-0.090*	0.108**	-0.102**	1.000					
(7) LLP_t-1_	0.116**	-0.006	-0.048	0.400***	-0.269***	0.507***	1.000				
(8) EMPSIZE_t-1_	0.323***	0.027	0.195***	0.909***	-0.506***	0.037	0.394***	1.000			
(9) GDP	0.039	-0.027	0.682***	-0.067	-0.125***	-0.133***	-0.118***	0.063	1.000		
(10) CPI	-0.119**	-0.020	-0.588***	-0.242***	0.226***	-0.031	-0.064	-0.177***	-0.391***	1.000	
(11) RIR	0.098*	-0.043	0.470***	0.201***	-0.109***	0.083*	0.082*	0.171***	0.026	-0.503***	1.000

This table reports the Pearson pairwise correlation coefficients of variables used in this study.

*** p < 0.01,

** p < 0.05,

* p < 0.1

From Table 1, ROA and ROE of Vietnamese banks have means of 1.275% and 12.526%, respectively, which clearly reflect the debt-based nature of the banking sector. Vietnamese banks’ NW_PROFIT is 10.367, suggesting that the average net profit per branch of Vietnamese banks is about VND 10.3 billion. Moreover, EMP_PROD has a mean of 0.256, meaning that an average bank employee generates VND 256 million profit per year for their bank.

## 4. Results and discussion

### 4.1. Baseline results

Table 3 reports the regression results of the baseline model. Columns 1 shows the estimation results of the bank profitability regressions while columns 2 present the results of employee productivity regressions. Columns 3 and 4 present the estimation results using ROA and ROE as the measure of bank profitability. We use different regression specifications to address whether our model is mis-specified: bivariate regression, the reduced-form regression with fixed effects, and full model regression.

**Table 3:** The relationship between anticorruption and bank profitability.

VARIABLES	(1)	(2)	(3)	(4)
	NW_PROFIT	EMP_PROD	ROA	ROE
ACINDEX_t-1_	2.8612***	0.0030	-0.0710	-0.4174
	(0.9216)	(0.0167)	(0.0870)	(0.7650)
BANKSIZE_t-1_	11.5689***	0.1910***	-0.3270*	-3.9548*
	(2.4130)	(0.0427)	(0.1659)	(1.9366)
ETA_t-1_	0.8221**	0.0110**	0.0563**	-0.1939
	(0.2917)	(0.0042)	(0.0216)	(0.1902)
NPL_t-1_	-1.0724**	-0.0322***	-0.0983***	-1.1423***
	(0.4553)	(0.0053)	(0.0245)	(0.2518)
LLP_t-1_	-0.8226	-0.0114	-0.1074	-1.7377
	(1.1350)	(0.0118)	(0.0937)	(1.3575)
EMPSIZE_t-1_	-6.9611***	-0.1534***	0.5415***	7.8545***
	(1.9859)	(0.0213)	(0.1639)	(2.0507)
GDP	-0.6581	-0.0019	0.0673	-0.1216
	(1.2229)	(0.0292)	(0.1577)	(1.7041)
CPI	-0.0306	0.0020	0.0121	0.1738
	(0.0644)	(0.0018)	(0.0106)	(0.1039)
RIR	0.0022	-0.0019*	-0.0002	-0.0111
	(0.0516)	(0.0009)	(0.0047)	(0.0413)
Bank FE	Yes	Yes	Yes	Yes
Standard errors clustered by	Year	Year	Year	Year
Observations	337	337	337	337
Adjusted R-squared	0.6269	0.6147	0.3829	0.2620

This table reports the regression results of bank performance on anticorruption index and control variables. The definitions of variables are provided in Table 1. Numbers in parentheses are robust standard errors.

*** p < 0.01,

** p < 0.05,

* p < 0.1.

We see that the coefficients of ACINDEX are positive and significant in Column 1, suggesting a positive association between average profitability per branch and anti-corruption intensity in the country. By standardizing coefficients, one standard deviation increase in anti-corruption intensity in Vietnam is associated with 11.8% increase in branch-level profitability, after controlling for bank-level fundamentals and macro-economic factors. However, we do not observe similar patterns with the coefficients of ACINDEX in employee productivity regressions in Table 3. Specifically, the coefficients of ACINDEX remain statistically insignificant in columns 2–4, implying that bank profitability does not significantly improve following an increase in anti-corruption intensity if we use conventional measures of bank profitability (e.g., ROA and ROE) or profit per employee. The findings jointly suggest evidence of increased bank profitability per branch following intensified anti-corruption in Vietnam while the overall profit does not immediately improve. This can be referred to as weak evidence of anti-corruption improving bank performance in Vietnam in the short-term, while it can be manifest in the longer-term which is tested in the later section of this paper. The empirical finding is different to that of Hoang et al. [5] and Kong et al. [1] that government’s anti-corruption exerts an immediate impact on the performance of non-financial firms. Similarly, the finding is different to that of Nobanee et al. [33] that bank-level anti-corruption immediately hinders bank performance. We attribute the difference in findings to the different sample choices: non-financial firms versus banks. Banks are large entities that are too big to immediately absorb the effect of anti-corruption as smaller non-financial firms, especially in the context of Vietnam.

Judging from the adjusted R-squares, the model explains 62.69% of the variations in average branch profitability (Column 1), 61.47% of the variations in EMP_PROD (Column 2), 38.29% of the variations of ROA (Column 4), and 26.2% of that of ROE (Column 5). The figures suggest that the using NW_PROFIT and EMP_PROD as the measures of bank profitability provide us better explanatory power than ROA and ROE.

### 4.2. Robustness and endogeneity treatments

To establish causality inference of the bank profitability–anti-corruption relationship, we address several issues that may confound the baseline findings: measurement errors, small sample bias, and causality.

First, we employ three alternative measurements of anti-corruption intensity using the composite indexes of anti-corruption with alternative datasets of news articles from three large online newspapers: Vnexpress, Dantri, and Nhandan. The three online newspapers have different ownership: Dantri (dantri.com.vn) is owned by a Vietnamese government agency, Nhandan (Nhandan.vn) is owned by the Communist Party of Vietnam, and Vnexpress is the largest Vietnamese private-owned online newspaper which belongs to FPT Corporation. By separating the three composite indexes, we can analyze and see if online newspaper ownership can confound the anti-corruption intensity measurement in our study. We substitute ACINDEX in Model (1) with each of the indexes: VNEINDEX, DTINDEX, and NDINDEX and re-estimate the model. The regression results are reported in Table 4.

**Table 4:** Alternative variable measurements.

*Panel A*. *ROA and ROE*						
VARIABLES	(1)	(2)	(3)	(4)	(5)	(6)
	ROA	ROE	ROA	ROE	ROA	ROE
VNEINDEX_t-1_	-0.1063	-0.7240				
	(0.0805)	(0.6908)				
DTINDEX_t-1_			-0.0370	-0.2722		
			(0.0910)	(0.8511)		
NDINDEX_t-1_					-0.0876	-0.2166
					(0.1099)	(0.9897)
BANKSIZE_t-1_	-0.3387*	-4.0509*	-0.3094*	-3.8515*	-0.3584*	-3.9728*
	(0.1686)	(1.9710)	(0.1618)	(1.9588)	(0.1868)	(2.0569)
ETA_t-1_	0.0562**	-0.1947	0.0565**	-0.1925	0.0556**	-0.1947
	(0.0217)	(0.1907)	(0.0214)	(0.1905)	(0.0221)	(0.1939)
NPL_t-1_	-0.0976***	-1.1384***	-0.0983***	-1.1435***	-0.0989***	-1.1415***
	(0.0242)	(0.2519)	(0.0245)	(0.2498)	(0.0249)	(0.2541)
LLP_t-1_	-0.1088	-1.7567	-0.1058	-1.7407	-0.1031	-1.6944
	(0.0952)	(1.3673)	(0.0929)	(1.3601)	(0.0948)	(1.3557)
EMPSIZE_t-1_	0.5510***	7.9394***	0.5317***	7.8147***	0.5427***	7.7825***
	(0.1635)	(2.0815)	(0.1671)	(2.0457)	(0.1592)	(2.0649)
GDP	0.0794	0.0019	0.0478	-0.1996	0.0697	-0.2674
	(0.1395)	(1.5210)	(0.1742)	(1.8540)	(0.1351)	(1.5459)
CPI	0.0137	0.1863*	0.0108	0.1664	0.0131	0.1700
	(0.0094)	(0.0966)	(0.0113)	(0.1056)	(0.0097)	(0.0997)
RIR	-0.0007	-0.0137	-0.0000	-0.0082	-0.0005	-0.0164
	(0.0044)	(0.0376)	(0.0055)	(0.0539)	(0.0042)	(0.0342)
Bank FE	Yes	Yes	Yes	Yes	Yes	Yes
Standard errors clustered by	Year	Year	Year	Year	Year	Year
Observations	337	337	337	337	337	337
Adjusted R-squared	0.3842	0.2628	0.3822	0.2619	0.3830	0.2617
*Panel B*. *Network profitability and employee productivity*						
VARIABLES	NW_PROFIT	EMP_PROD	NW_PROFIT	EMP_PROD	NW_PROFIT	EMP_PROD
VNEINDEXt-1	2.7633***	-0.0024				
	(0.8567)	(0.0157)				
DTINDEXt-1			1.8121*	0.0001		
			(0.8383)	(0.0161)		
NDINDEXt-1					2.5557*	0.0039
					(1.2290)	(0.0222)
BANKSIZE	11.6487***	0.1896***	10.9150***	0.1903***	12.3081***	0.1925***
	(2.4988)	(0.0430)	(2.3967)	(0.0409)	(2.8460)	(0.0478)
ETA	0.8240**	0.0110**	0.8201**	0.0110**	0.8498**	0.0110**
	(0.2974)	(0.0042)	(0.2933)	(0.0041)	(0.3044)	(0.0043)
NPL	-1.0981**	-0.0322***	-1.0560**	-0.0322***	-1.0360**	-0.0321***
	(0.4509)	(0.0053)	(0.4594)	(0.0053)	(0.4424)	(0.0052)
LLP	-0.9339	-0.0121	-0.7681	-0.0118	-1.1112	-0.0116
	(1.1207)	(0.0121)	(1.1343)	(0.0112)	(1.1130)	(0.0125)
EMPSIZE	-6.9111***	-0.1518***	-6.7025***	-0.1526***	-6.6972***	-0.1535***
	(1.9803)	(0.0220)	(1.9725)	(0.0198)	(2.0845)	(0.0233)
GDP	-0.3595	0.0012	-0.0526	-0.0001	-0.2887	-0.0021
	(1.1306)	(0.0267)	(1.2221)	(0.0309)	(1.0123)	(0.0247)
CPI	-0.0477	0.0021	0.0186	0.0020	-0.0412	0.0019
	(0.0696)	(0.0017)	(0.0704)	(0.0018)	(0.0900)	(0.0018)
RIR	0.0352	-0.0018**	-0.0164	-0.0019	0.0246	-0.0019**
	(0.0429)	(0.0008)	(0.0683)	(0.0012)	(0.0464)	(0.0007)
Bank FE	Yes	Yes	Yes	Yes	Yes	Yes
Standard errors clustered by	Year	Year	Year	Year	Year	Year
Observations	288	337	288	337	288	337
Adjusted R-squared	0.6257	0.6147	0.6246	0.6147	0.6235	0.6147

This table reports the regression results of bank performance on anti-corruption indexes using ROA and ROE as the alternative dependent variables. Definitions of variables are in Table 1. Numbers in parentheses are robust standard errors. *** p < 0.01, ** p < 0.05, * p < 0.1.

This table reports the regression results of bank performance on anti-corruption indexes using bank network profitability and employee productivity as the alternative dependent variables. Definitions of variables are in Table 1. Numbers in parentheses are robust standard errors. *** p < 0.01, ** p < 0.05, * p < 0.1.

Panel A, Table 4, shows that the coefficient of VNEINDEX, DTINDEX, and NDINDEX remain statistically insignificant in all regression specifications using ROA and ROE alternatively as the dependent variable. The patterns are consistent with that in the ROA and ROE regression in Table 3. In Panel B, we document similar pattern of the anti-corruption–average branch profitability: the coefficient of VNEINDEX, DTINDEX and NDINDEX are positive and statistically significant in branch profitability regressions in columns 1, 3, and 5, Panel B of Table 4. On the other hand, the coefficients of the anti-corruption variables remain insignificant in employee profitability regressions in columns 2, 4, and 6, Table 4. Hence, the robustness test results corroborate our baseline findings.

Second, our sample covers Vietnamese commercial banks from 2005–2019, thus the sample size is small despite we include most banks with available data in this study. An issue of small samples is that the findings might not be applicable to a large sample, therefore, has limited contributions. To overcome this limitation and generalize the findings from this study, we re-estimate the baseline model using bootstrapped standard errors with 100,000 replications. Bootstrap is an intensive computer technique of random resampling with replacement, that allows to control and check the stability of estimation results. Generally, bootstrap is asymptotically more accurate than the standard estimations under the assumption of normality [41] which is quite difficult to achieve with small samples. We report the bootstrap estimation results (100,000 replications) of Model (1) in columns 1–2, Table 5. Similar results compared to the baseline results are documented when we use 1,000 and 10,000 replications for the bootstrap estimations. The results bolster our confidence that small sample bias does not undermine the validity of our findings.

**Table 5:** Further robustness checks.

VARIABLES	Bootstrapping standard errors (100,000 replications)				2SLS/IV regression	
	(1)	(2)	(3)	(4)	(5)	(6)
	NW_PROFIT	EMP_PROD	ROA	ROE	First stage ACINDEX	Second stage NW_PROFIT
ACINDEX_t-1_	2.9722**	0.0365*	-0.0710	-0.4174		26.0773**
	(1.4026)	(0.0189)	(0.0952)	(0.8662)		(11.9347)
ELECTION					0.1808***	
					(0.0558)	
BANKSIZE_t-1_	11.5689***	0.1921***	-0.3270**	-3.9548***	-0.1917*	15.9485***
	(3.6403)	(0.0339)	(0.1560)	(1.5028)	(0.1159)	(4.8319)
ETA_t-1_	0.8221***	0.0111***	0.0563***	-0.1939	0.0063	0.6706**
	(0.1864)	(0.0029)	(0.0171)	(0.1405)	(0.0093)	(0.3129)
NPL_t-1_	-1.0724*	-0.0322***	-0.0983***	-1.1423***	0.0075	-1.2304*
	(0.6076)	(0.0069)	(0.0314)	(0.3097)	(0.0268)	(0.7416)
LLP_t-1_	-0.8226	-0.0114	-0.1074	-1.7377	-0.1324*	2.3505
	(1.6664)	(0.0167)	(0.1054)	(1.4625)	(0.7895)	(2.4165)
EMPSIZE_t-1_	-6.9611*	-0.1534***	0.5415**	7.8545***	0.2874**	-13.8047**
	(3.9804)	(0.0385)	(0.2175)	(2.3108)	(0.1434)	(6.1071)
GDP	-0.6581	-0.0019	0.0673	-0.1216	0.6253***	-14.9998**
	(1.3018)	(0.0161)	(0.0889)	(0.9165)	(0.0440)	(6.8894)
CPI	-0.0306	0.0019	0.0121	0.1738	0.0169*	-0.5615
	(0.0955)	(0.0016)	(0.0105)	(0.1167)	(0.0101)	(0.3992)
RIR	0.0022	-0.0017	-0.0002	-0.0111	0.0103***	-0.3929*
	(0.0661)	(0.0012)	(0.0094)	(0.1186)	(0.0031)	(0.2033)
Bank FE	Yes	Yes	Yes	Yes		Yes
Observations	288	337	337	337		337
Adjusted R-squared	0.6269	0.6147	0.3829	0.2620		0.5102
Under-identification test statistic						7.768
Weak identification test statistic						10.509
Anderson-Rubin confidence interval						[-20.706, 72.860]

This table reports the robustness tests’ regression using bootstrapping technique and the instrumental variable estimator. Definitions of variables are in Table 1. Numbers in parentheses are robust standard errors.

*** p < 0.01,

** p < 0.05,

* p < 0.1.

Last but not least, we employ the instrumental variable approach to establish causal inference of the newfound relationship between bank profitability and anti-corruption intensity in Vietnam. We use an election year dummy as the instrumental variable for anti-corruption intensity, as timing of scheduled elections matter to anti-corruption activities [19]. Intuitively, scheduled elections do not directly affect bank profitability and therefore satisfies the excluded restrictions for the Two-stage Least Square/ Instrumental variable (2SLS/IV) regression. We report the 2SLS/IV regression results of Model (1) in columns 3–4, Table 5. The second-stage results show that the coefficient of the instrumented ACINDEX is positive and statistically significant, suggesting a causal relationship between anti-corruption intensity and bank profitability.

To summarize, after employing different econometric tests, we suggest that the anti-corruption campaign exerts a significant impact on average branch profitability of Vietnamese commercial banks.

### 4.3. The evolution of the effects

**Fig 2:**
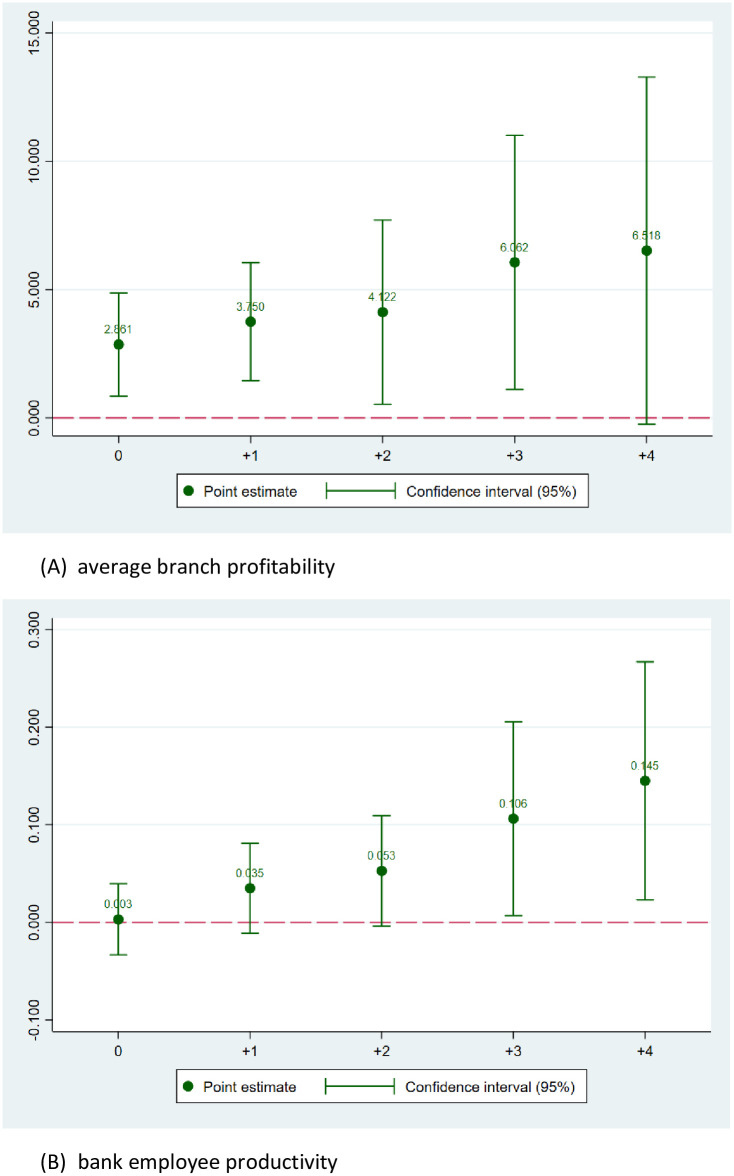
The time variations of the effect of anticorruption on branch profitability and employee productivity. (A) average branch profitability. (B) bank employee productivity.

**Fig 3:**
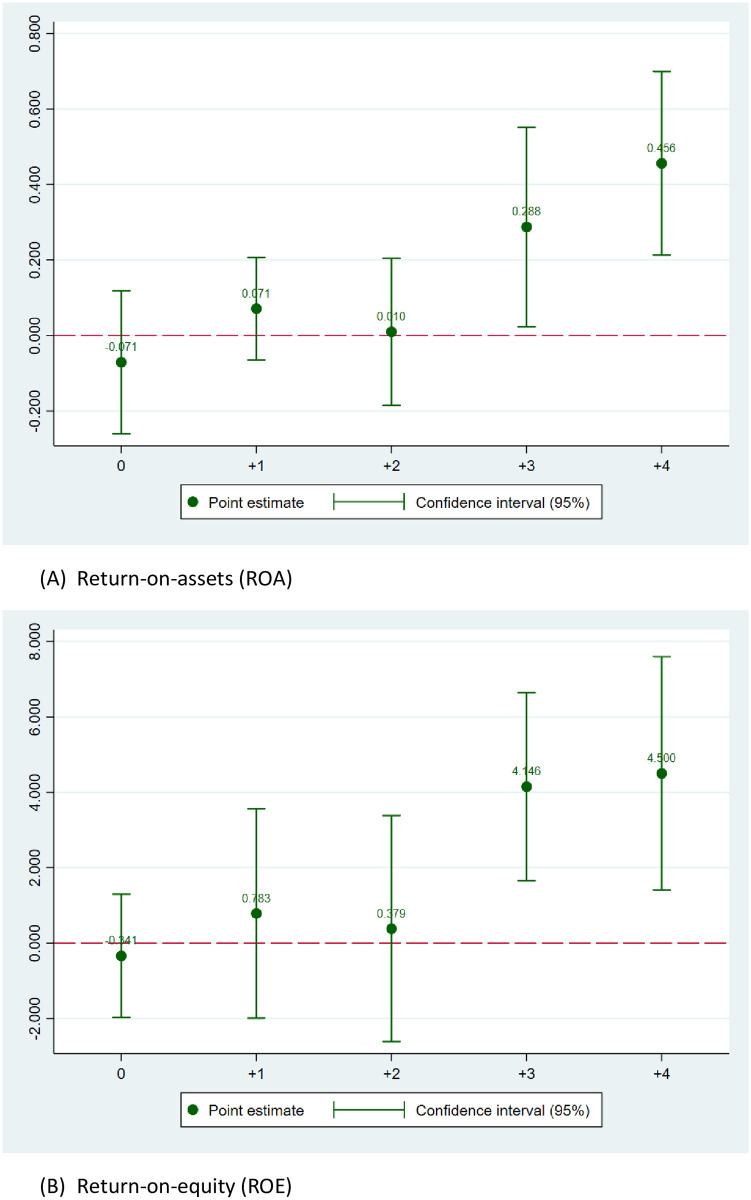
The time variations of the effect of anticorruption on bank ROA and ROE. (A)Return-on-assets (ROA). (B) Return-on-equity (ROE).

Economic and reform policies usually manifest their effects in the long term rather than immediately. In this section, we take one step further to address the evolution of the effect of anti-corruption on bank profitability in the time dimension. As the economic impacts of anti-corruption might be time-variant [42], it is important to examine how the effects of anti-corruption on different types of bank profitability vary in the time dimension. We re-estimate Model (1) for using different timing differences between the outcome variables (NW_PROD, EMP_PROD, ROA, and ROE, respectively) relative to the variable of interest (i.e., ACINDEX) and see how the effects change over time. For better illustration of the results, we plot the coefficients of ACINDEX and illustrate the evolution of the effects of anti-corruption on profitability of banks in Figs 2 and 3.

One the one hand, the coefficient plots in Fig 2A show that the effect of anti-corruption on average branch profitability lasts for the 4-year period. On the other hand, Fig 2B shows that bank employee profitability improves with more anti-corruption efforts in the long run, as the coefficient of ACINDEX in EMP_PROD regressions gradually turn statistically significant and positive since year t+2. For better illustration of the results, Fig 3 demonstrate the evolution of anti-corruption’s effect on bank ROA and ROE over time. Similar patterns are documented when we respectively use ROA and ROE as the measure of profitability [43]. The plots of regression coefficients suggest a lagged effect of institutional changes (i.e., the anti-corruption campaign) on bank performance in Vietnam. Although the effect only shows immediately at the average branch-level and employee profitability, it gradually become significant in the longer windows, which is consistent with the general lagged effect of public policies [44] and extend the findings of the previous literature on how anti-corruption impacts non-financial firms’ performance [1, 5].

The findings suggest the long-term effect of the institutional reform in the country on overall bank profitability, which is in line with government policies generally have a lag effect of two years or more [45, 46]. Our finding on the evolution of the effect is robust to different measurements of bank profitability. The finding further corroborates the notion that anti-corruption enhances the institutions, business environment, and setup motivation for future economic growth [3, 6, 47, 48]. In other words, the anti-corruption activities in Vietnam are not simply a political game of political factions but have an evident positive economic impact on the banking sector in Vietnam. These finding complements that of Hoang et al. [5] when they suggest the positive effect of the anti-corruption campaign of Vietnam on non-financial firms’ profitability.

In summary, we show that the effect of anti-corruption on bank profitability is not short-term but strongly manifests in the long-term.

## 5. Conclusion

In this study, we show that anti-corruption has a positive impact on the profitability of Vietnamese commercial banks, however, it manifests in the long term. The empirical findings suggest that the impact of anti-corruption on bank performance turns favourable after two years, which is similar to the general policy lag [45]. The finding demonstrates the bright side of the anti-corruption campaign in Vietnam; it is not solely politics and seems to foster economic growth in the banking sector in Vietnam, therefore having a significant and positive impact on the economy. A caution note is that our findings do not necessarily suggest the motives of the anti-corruption campaign in the country, but rather focus on the consequences of the institutional change.

Our study has implications for public policy and bank strategy in the unique context of Vietnam. Branch structure is important in times of changing institutions as it may improve bank efficiency [49]. Specifically, Vietnamese commercial banks may need to consider profit maximization at the branch-level. Increasing anti-corruption generally lead to a higher turnover of government officials [1], thus indirectly generating more policy uncertainty. Under such uncertainty associated with anti-corruption activities, commercial banks may need to find an optimal branch structure to maintain resilience in the short-term while waiting for the institutions and the business environment change for the better in the long-term. The findings of this study imply that anti-corruption in Vietnam seems to foster economic growth in the banking sector in the long run but not immediately, hence, providing an academic reference for future public policy about the economic impact of the cracking down on corruption in the country. It is important to observe during a long window to see the dynamic impact of anticorruption on the banking sector. Therefore, investors in Vietnam’s financial market may need to pay close attention to the developments of the anti-corruption campaign in the country to evaluate its impact on the performance of their investment.
